# Sex and limb comparisons of neuromuscular function in the morning versus the evening

**DOI:** 10.14814/phy2.15260

**Published:** 2022-05-17

**Authors:** Garrett R. Augsburger, Alisa Soloveva, Joshua C. Carr

**Affiliations:** ^1^ Kinesiology Department Texas Christian University Fort Worth Texas USA; ^2^ Department of Medical Education Texas Christian University School of Medicine Fort Worth Texas USA

**Keywords:** diurnal, EMG, force steadiness, RER, RFD, sex differences, time of day

## Abstract

The time‐of‐day influence on neuromuscular function is well‐documented, but important details remain elusive. It is currently unknown whether males and females differ in their diurnal variation for optimal neuromuscular performance. The purpose of this study is to identify the time‐of‐day influence on neuromuscular function between sexes and determine whether these responses differ for the upper versus lower limbs. A group of males (*n* = 12) and females (*n* = 15) completed neuromuscular performance testing in the morning (07:00–09:00) and evening (17:00–19:00) on separate days in a randomized order. Maximal force, the normalized rate of force development, EMG, normalized EMG rise, and submaximal force steadiness were compared between morning and evening hours. The main findings show that maximal force was greater in the evening for the knee extensors (*d* = 0.570, *p* < 0.01) but not the elbow flexors (*d* = 0.212, *p* = 0.281), whereas maximal muscle excitation was greater in the evening for the biceps brachii (*d* = 0.348, *p* < 0.01) but not the vastus lateralis (*d* = 0.075, *p* = 0.526) with no influence of sex. However, force steadiness during knee extension was superior in the evening versus the morning for males (*d* = 0.734, *p* = 0.025) and compared to evening values for females (*g* = 1.19, *p* = 0.032). Overall, these findings show that time‐of‐day affects the knee extensors more than the elbow flexors and that diurnal variability between sexes appears to be task‐dependent.

## 1 INTRODUCTION

Circadian rhythms produce endogenous variations in physiological outputs that influence physical performance across a 24‐h cycle (Lok et al., 2020). These circadian rhythms exist in virtually every physiological measure of performance and are controlled by a ‘brain clock’ located in the suprachiasmatic nucleus of the hypothalamus (Bailey & Silver, 2014; Douglas et al., 2021; Kuljis et al., 2013). Time‐dependent variations in maximal performance during waking hours have been documented in laboratory settings (Gauthier et al., 1996; Guette et al., 2005a; Sedliak et al., 2008) and competitive sports events (Lok et al., 2020). Evidence exists for alterations in neural excitation (Gueldich et al., 2016; Lang et al., 2011; Martin et al., 1999), neurohormonal flux (Chtourou et al., 2012; Racinais et al., 2005; Tamm et al., 2009), contractile alterations at the muscle fiber level (Mirizio et al., 2020; Sedliak et al., 2008), and some recent hypotheses implicate circadian clock genes (Douglas et al., 2021; Dyar et al. 2012). Compelling evidence shows that maximal strength peaks in the evening (~16:00–20:00 h) and is lowest in the morning (~06:00–10:00 h) (Hatfield et al., 2016; Mirizio et al., 2020; Sedliak et al., 2008). The implications of the circadian influence on physical performance extend from standardizing laboratory conditions (Nuzzo et al., 2018) to optimizing training interventions in healthy (Küüsmaa et al., 2016) and diseased (Mancilla et al., 2021; Savikj et al., 2019) populations. Despite the well‐documented evening superiority in maximal strength, additional features of time‐of‐day‐dependent changes in neuromuscular function are not as clear.

The influence of time of day on maximal isometric force has been documented across many muscle groups (Drust et al., 2005; Gauthier et al., 1996; Küüsmaa et al., 2016). There is some evidence (Guette et al., 2005a; 2005b, Knaier et al., 2019) that different muscle groups may respond uniquely in the morning versus evening, yet direct comparisons between limbs are lacking. Since the muscles of the lower limb are involved in locomotor activity throughout the day, it may be that ambulation provides a passive warmup effect not shared by the muscles of the upper limb. Moreover, additional force and electromyographic (EMG) metrics that are strongly related to neural input are not as well described in the literature. The rate of force development (RFD) and EMG rise (RER) are heavily influenced by the volitional neural drive at contraction onset (Andersen & Aagaard, 2006; Del Vecchio et al., 2018; Klass et al., 2008). The time‐dependent rise of these metrics during rapid contractions allows inferences on the magnitude of motor unit activation during the initial phase (<75 ms) of contraction, whereas intrinsic properties of the activated muscle are more dominant in the later phases (>150 ms)(Andersen & Aagaard, 2006; Maffiuletti et al., 2016). Additionally, measurements of force steadiness provide another view of motor unit activity since force fluctuations are influenced by the common oscillations in synaptic input delivered to the active alpha motor neurons (Brown et al., 2010; Harwood et al., 2010; Inglis & Gabriel, 2021; Jakobi et al., 2018; Taylor et al., 2003). The intrinsic input‐output properties within force steadiness measurements therefore provide a general indication of the stability of descending neural drive (Dideriksen et al., 2012). Compared to maximal isometric force, these variables are underrepresented in the literature and may provide additional inferences relating to the neural contributions to the time‐of‐day effect on neuromuscular function. A series of recent experiments have shown that females demonstrate greater force variability than males during low‐force isometric contraction (Brown et al., 2010; Jakobi et al., 2018). Importantly, the vast majority of the published literature regarding the time‐of‐day influence on neuromuscular function has focused on males, with very little data available on how neuromuscular function varies across the day in female participants (Bambaeichi et al., 2004; Birch & Reilly, 2002; Giacomoni et al., 2005).

The extent to which males and females differ in their susceptibility to the influence that time‐of‐day exerts on neuromuscular function is largely unknown (Giacomoni et al., 2005). This information is important as identifying sex‐based differences in diurnal patterns could further optimize rehabilitation and training practices. Similarly, examining whether muscle groups of different structure and function exhibit unique time‐of‐day responses has broad implications (i.e., precision rehabilitation, testing, training). The purpose of this study is to identify the time‐of‐day influence on neuromuscular function in the upper versus lower limbs and determine whether biological sex moderates the time‐of‐day effects. We hypothesized that the knee extensors would show greater time‐of‐day effects than the elbow flexors and that females would have a greater time‐of‐day variation in the outcome variables than males (Bambaeichi et al., 2004; Birch & Reilly, 2002).

## 2 MATERIALS AND METHODS

### 2.1 Participants

An a priori power analysis was performed based on the effect sizes of morning versus evening isometric forces for the elbow flexors (Gauthier et al., 1996; Guette et al., 2005a) and knee extensors (Giacomoni et al., 2005). The power analysis was performed as described by Beck (2013) for a within‐between interaction with α set at 0.05, power set at 0.80, two groups, two measurements, and an effect size of (0.30). The power analysis computed 24 participants for adequate statistical power and a total of 29 participants were enrolled in the study with 27 completing all visits. The participants reported an absence of injury and disease and provided written informed consent before participation in the study. The participant demographics are shown in Table 1. A menstrual status questionnaire was completed by the female participants, eight of whom reported hormonal contraceptive use, while the others indicated eumenorrhea. Of those, it was indicated that the majority (*n* = 5) completed testing during the luteal phase while some (*n* = 2) completed testing during the follicular phase based on the questionnaire. All participants self‐reported they were physically active at the time of their enrollment. The average resistance training experience was ~6 years for males and ~4 years for females. All procedures were approved by the Institutional Review Board for Human Subjects at Texas Christian University (IRB#1920‐342).

**TABLE 1:** Participant demographics along with self‐reported sleep time the night before morning (AM) and evening (PM) testing visits

		Female *n* = 15	Male *n* = 12
Age (yr)		23 (3)	24 (3)
Height (cm)		166.6 (7.1)	177.5 (5.1)
Weight (kg)		66.1 (16.8)	87.8 (15.5)
Sleep (hr)	AM	7.5 (1.2)	6.9 (1.1)
	PM	7.1 (1.3)	7.4 (0.9)
Hormonal contraceptives		*n* = 8	
Menstrual phase of testing		Follicular *n* = 2 Luteal *n* = 5	

### 2.2 Experimental design

This study used a crossover design to determine whether neuromuscular function differs between the morning (07:00–09:00) versus the evening (17:00–19:00) hours. Before the experimental visits, participants completed a familiarization session that was randomized to either the morning or the evening hours of testing. The participants practiced each of the experimental tasks ~3× for the upper and lower limb. The participants were instructed to refrain from exercise 24–48 h before visiting the laboratory and maintain normal sleep and nutrition routines. A primary aim was to determine whether sex influences the time‐of‐day effects on neuromuscular function. A secondary aim was to examine the influences of time of day on the neuromuscular function between the large muscle groups of the upper versus lower limbs. Maximal voluntary contraction (MVC) force, normalized RFD (nRFD), maximal EMG amplitude, the normalized rate of rise of the EMG (nRER) signal, and submaximal force steadiness were used to quantify neuromuscular function. Force testing was performed on the elbow flexors and knee extensors in a randomized order with the corresponding surface EMG responses collected from the biceps brachii and vastus lateralis. The experimental visits were 1 h in duration and were completed 3–7 days between sessions.

### 2.3 Experimental procedures

#### 2.3.1 Isometric force

Isometric force of the knee extensors and elbow flexors were measured in custom‐made testing apparatuses with a tension‐compression load cell (SSM‐500, Interface Inc., Scottsdale, AZ.). For the knee extension assessment, the participant was seated on a knee extension machine with their hip and knee angles positioned at 90°. For elbow flexor assessment, the shoulder and elbow angles were placed at 90° with a goniometer while the participant was in a seated position. A cuff attached to the load cell was placed around the wrist of the participant to measure elbow flexion force. The force testing procedures were the same for the knee extensors and elbow flexors. Specifically, a series of submaximal isometric contractions were performed to warm up the respective joint by performing a 3‐s isometric contraction at 25%, 50%, 75%, and 90% of their perceived maximal force with 1 min of recovery between attempts. Following the warm‐up, maximal force and RFD were assessed under two conditions (i.e., “*fast*” and “*hard*”*)*, as suggested by Maffiuletti et al. (2016). The first series consisted of three fast, ballistic intent maximal contractions. The participant was instructed to exert their maximal force as rapidly as possible with ballistic intent. Each contraction was 1‐s in duration with 1 min of recovery between attempts. For the second series of maximal contractions, the participant was instructed to exert their maximal force as hard as possible with maximum force intent. Each contraction was performed three times and was 3‐s in duration with 1 min of recovery between attempts. Similarly, the RER and maximal EMG amplitude values were quantified from the *fast* and *hard* contractions, respectively. In total, six maximal contractions were performed for each muscle group, the results of which were averaged within conditions (i.e., *fast* and *hard*) for statistical analysis.

#### 2.3.2 Submaximal force steadiness

Following the determination of isometric force, submaximal force steadiness was measured with a trapezoidal force matching task at 30% MVC. The participants received real‐time force feedback displayed on a 32” TV screen that was approximately 1 meter in front of them at eye level and were instructed to match their force output as closely as possible to the template. The participants increased their force output at a rate of 10% MVC/s, held as steadily as possible at the 30% MVC target force for 9 s, and then decreased their force output at a rate of 10% MVC/s. This task was performed 3 times for each muscle group. The coefficient of variation (CoV) was used to quantify force steadiness during the plateau of the force‐matching task. The coefficient of variation was calculated for each submaximal force‐matching task and was averaged for the respective muscle group and visit for statistical analysis. The first and final 0.5 s of the plateau were not included in the force steadiness analyses given the changes brought about by the rising and falling force tracing, as a result, only the mean of the 8 s plateau during the submaximal contraction was used to determine submaximal force steadiness.

#### 2.3.3 Instrumentation and signal processing

The EMG signals were obtained with Trigno^TM^ wireless bipolar surface EMG sensors (interelectrode distance =10 mm, 37 mm × 26 mm × 15 mm; Delsys Inc., Natick, MA, USA). The surface electrodes were placed over the vastus lateralis and biceps brachii muscles of the dominant leg and arm following international standards for surface EMG (Hermens, 2000). Specifically, the placement of the EMG electrode on the vastus lateralis was at ~66% distance between the anterior superior iliac spine and lateral aspect of the patella. For the biceps brachii, the electrode was placed approximately halfway between the acromion process and the antecubital space at the peak of the muscle belly. Before sensor placement, the sensor pick‐up area was shaved and cleaned with alcohol swabs to remove hair and debris. The electrode sites were outlined with a waterproof felt‐tip pen for replication. Both the force and EMG signals were sampled at 1926 k Hz and were stored on a laptop computer (Intel Core i7 8th generation). Custom software (LabVIEW, National Instruments, Austin, TX, USA) was used for the force and EMG signal processing. The force signal was smoothed with a 25 ms zero‐shift moving average and the EMG signals were pre‐amplified and bandpass filtered (20–450 Hz) with a 25 ms zero‐shift moving RMS. The onsets of force and EMG were visually determined by placing cursors around the regions of interest and magnifying the scale of their time curves in separate plots. The force and EMG onsets were identified as the point at which the signal deflected 2 SD away from baseline values. The MVC force was selected from the highest 500 ms mean value during the plateau of the *hard* MVC. The amplitude of the EMG signal was quantified as the highest 100 ms root mean square value of the EMG signal during the *hard* MVC. RFD was determined from the linear slope of the force‐time curve at time intervals of 0–50 (nRFD_50_), 0–100 (nRFD_100_) ms, and the peak rate of change in the force‐time curve (nRFD_peak_) from force onset of the *fast* MVC and were normalized against the maximal force value obtained during the hard MVC. The RER was quantified from the linear slope of the EMG‐time curve at intervals of 0–30 (nRER_30_) and 0–50 (nRER_50_) ms from EMG onset of the *fast* MVC and were normalized against the maximal EMG value obtained during the hard MVC. Unpublished test‐retest intersession reliability from our laboratory shows good‐excellent reliability for assessments of maximal isometric force (ICC_2,1_ > 0.95, SEM% = ~3.0%) and maximal EMG RMS (ICC_2,1_ > 0.95, SEM% = ~8.0%).

### 2.4 Statistical analysis

Data are presented as means ± standard deviations. Separate three‐way (time‐of‐day [morning, evening] × sex [male, female] × limb [elbow flexor, knee extensor]) mixed factorial analysis of variance (ANOVA) tests were used to analyze the force and EMG data. Significant interactions and main effects were followed up with simple effects tests and Holm‐Bonferroni pairwise comparisons, respectively. The Shapiro‐Wilk test was used to determine normal distribution of the data and Levene’s test was used for homogeneity of variances between groups. The partial eta squared (η_p_^2^) statistic is provided for all repeated measures ANOVAs, with values of 0.01, 0.06, and 0.14 corresponding to small, moderate, and large effects, respectively (Stevens, 2007). Additionally, Cohen’s *d* and Hedge’s *g* were computed to interpret the effect size for specific mean comparisons of interest with traditional values of 0.20, 0.50, and 0.80 corresponding to small, moderate, and large effects, respectively (Cohen, 1988). SPSS software (IBM, Version 26) was used for statistical analysis and alpha was set at 0.05.

## 3 RESULTS

### 3.1 Maximal voluntary contraction force and EMG amplitude

**FIGURE 1:**
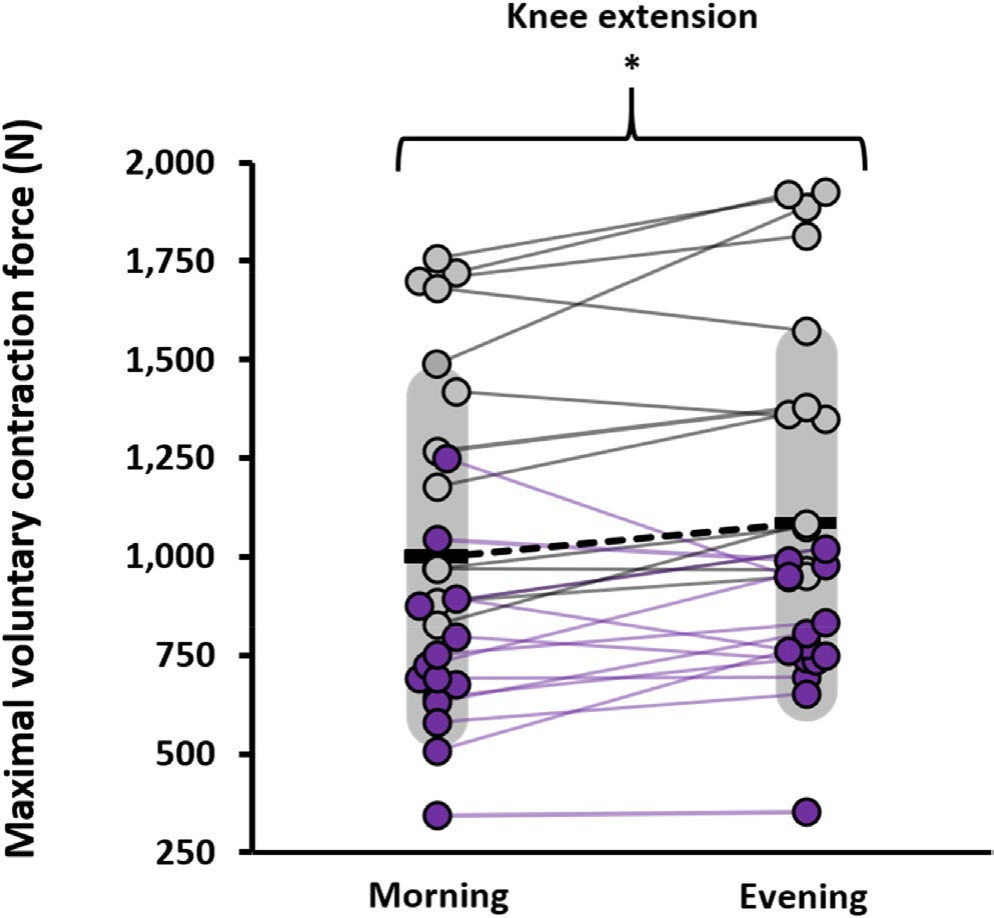
Plots of maximal isometric force values during knee extension in the morning (07:00–09:00 h) and the evening (17:00–19:00 h) for each individual. The darker circles and lines reflect females (*n* = 15), and the lighter circles and lines reflect males (*n* = 12). Means are represented by the horizontal black bar and variability (SD) by the grey shading. *Significant (*p* < 0.01, *d* = 0.570) difference between morning and evening

**FIGURE 2:**
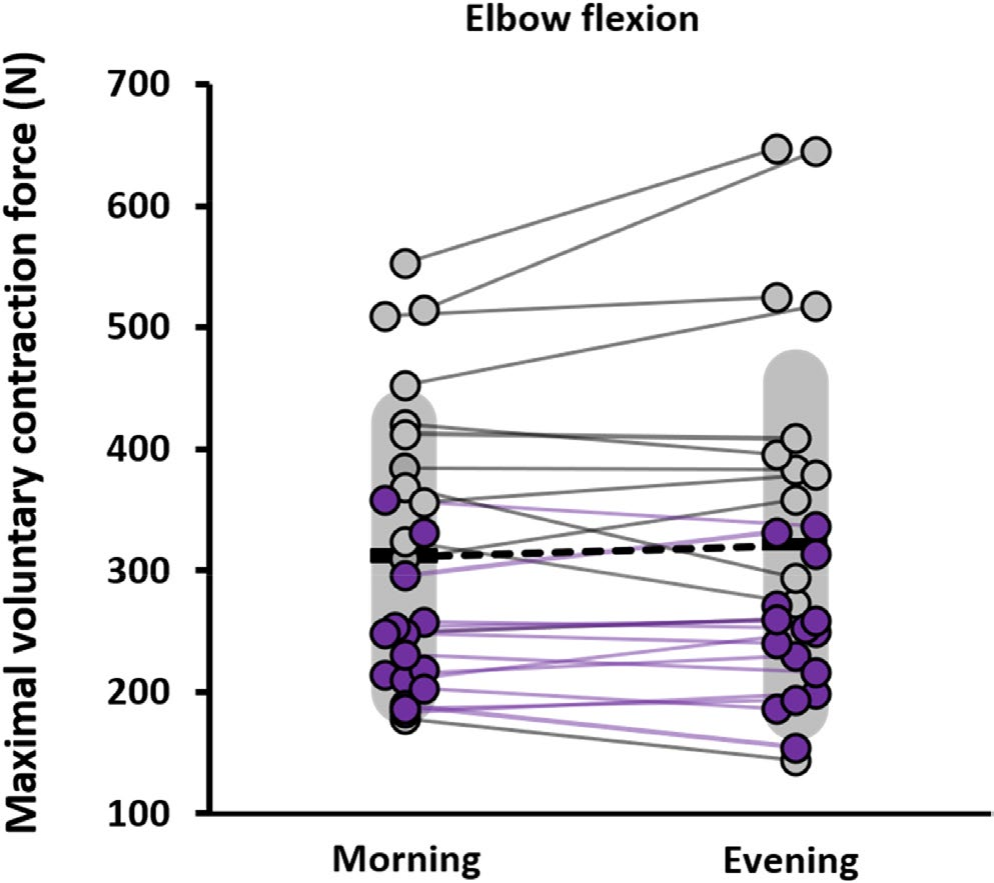
Plots of maximal isometric force values during elbow flexion in the morning (07:00–09:00 h) and the evening (17:00–19:00 h) for each individual. The darker circles and lines reflect females (*n* = 15), and the lighter circles and lines reflect males (*n* = 12). Means are represented by the horizontal bar and variability (SD) by the grey shading

**FIGURE 3:**
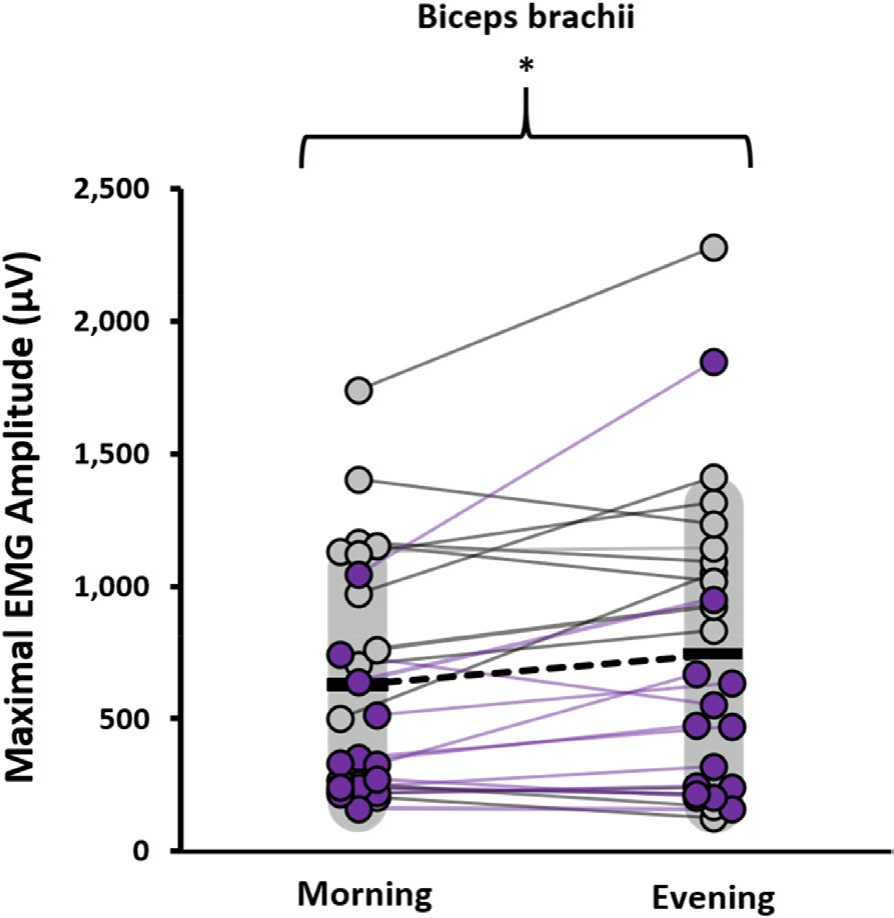
Plots of maximal EMG amplitude during elbow flexion in the morning (07:00–09:00 h) and the evening (17:00–19:00 h) for each individual. The darker circles and lines reflect females (*n* = 15), and the lighter circles and lines reflect males (*n* = 12). Means are represented by the horizontal bar and variability (SD) by the grey shading. *Significant (*p* < 0.01, *d* = 0.348) difference between morning and evening

The analysis revealed a significant time‐of‐day × limb interaction (*p* = 0.016, η_p_^2^ = 0.211, F_1,25_ = 6.69). Simple effects tests showed that maximal force was significantly greater in the evening for the knee extensors (*p* < 0.01, *d* = 0.570, Figure 1), but not the elbow flexors (*p* = 0.281, *d* = 0.212, Figure 2). There was no time‐of‐day ×sex interaction for maximal force (*p* = 0.249, η_p_^2^ = 0.053, F_1,25_ = 1.39) or EMG amplitude (*p* = 0.989, η_p_^2^ < 0.01, F_1,25_ < 0.01). The analysis revealed a significant time of day × limb interaction for the maximal EMG amplitude values (*p* = 0.020; η_p_^2^ = 0.197; F_1,25_ = 6.143) with simple effects tests showing the biceps brachii exhibiting greater values in the evening versus the morning (*p* < 0.01; *d* = 0.348, Figure 3) while no effect was shown for the vastus lateralis (*p* = 0.526, *d* = 0.075).

### 3.2 Rate of force development and rate of EMG rise

The analysis showed no time‐of‐day × sex interaction for nRFD_50_ (*p* = 0.678, η_p_^2^ < 0.01, F_1,25_ = 0.176), nRFD_100_ (*p* = 0.762, η_p_^2^ < 0.01, F_1,25_ = 0.094), or nRFD_peak_ (*p* = 0.826, η_p_^2^ < 0.01, F_1,25_ = 0.050) and no main effects for time‐of‐day for any nRFD variable (*p* > 0.216) related to our hypotheses. Table 2 summarizes the results for nRFD. The analysis showed no time‐of‐day × sex interaction for nRER_30_ (*p* = 0.329, η_p_^2^ < 0.01, F_1,25_ = 0.992) or nRER_50_ (*p* = 0.055, η_p_^2^ < 0.139, F_1,25_ = 4.05) and no main effects for time‐of‐day for any nRER variable (*p* > 0.641) related to our hypotheses. Table 3 summarizes the results for nRER.

**TABLE 2:** Means (SD) for normalized rate of force development during ballistic intent maximal voluntary contractions of the knee extensors and elbow flexors in the morning (AM) and evening (PM). The values for the male (n = 12) and female (n = 15) groups are shown individually and combined

	nRFD_50_			nRFD_100_			nRFDpeak		
	AM	PM		AM		PM	AM	PM	
Knee extension rate of force development									
Male	824 (749)	703 (544)		595 (183)		544 (142)	2975 (1778)	2710 (1491)	
Female	267 (237)	235 (157)		395 (219)		413 (204)	1269 (1017)	1082 (539)	
Combined	515 (589)	443 (441)		484 (224)		471 (188)	2028 (1625)	1805 (1333)	
Elbow flexion rate of force development									
Male	318 (250)		400 (284)		469 (266)	574 (176)	1542 (1232)		1883 (1248)
Female	99 (52)		159 (199)		190 (139)	259 (287)	737 (256)		848 (482)
Combined	197 (201)		266 (265)		314 (245)	399 (288)	1095 (918)		1308 (1029)

**TABLE 3:** Means (SD) for normalized rate of EMG rise during ballistic intent maximal voluntary contractions of the knee extensors and elbow flexors in the morning (AM) and evening (PM). The values for the male (*n* = 12) and female (*n* = 15) groups are shown individually and combined

	nRER_30_		nRER_50_	
	AM	PM	AM	PM
Vastus lateralis rate of EMG rise				
Male	392 (253)	351 (306)	497 (339)	403 (293)
Female	336 (198)	350 (183)	305 (175)	359 (218)
Combined	361 (222)	350 (240)	390 (273)	379 (250)
Biceps brachii rate of EMG rise				
Male	365 (270)	342 (524)	397 (213)	326 (195)
Female	256 (209)	312 (342)	194 (159)	243 (256)
Combined	305 (240)	326 (424)	285 (209)	280 (230)

### 3.3 Submaximal force steadiness

**FIGURE 4:**
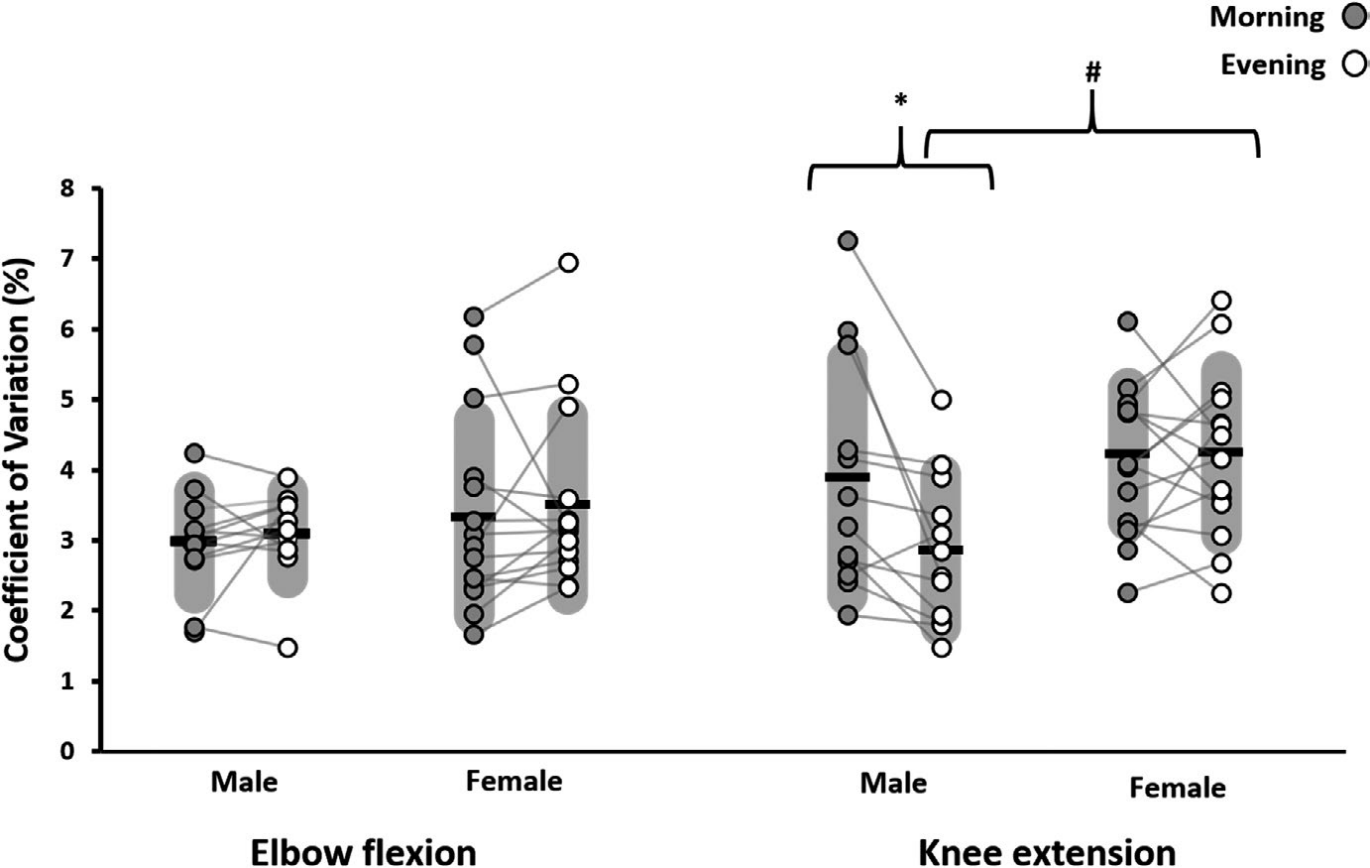
Plots of the coefficients of variation during steady submaximal isometric contractions of the elbow flexors and knee extensors in the morning (07:00–09:00 h) and the evening (17:00–19:00 h) for males (*n* = 12) and females (*n* = 15). Means are represented by the horizontal bar and variability (SD) by the grey shading. *Significant (*p* = 0.025, *d* = 0.734) difference between morning and evening, ^#^significant (*p* = 0.032, *g* = 1.19) sex difference in the evening

The analysis on force steadiness revealed a time‐of‐day × sex interaction (*p* = 0.035, η_p_^2^ = 0.166, F_1,25_ = 4.96), a time‐of‐day × limb interaction (*p* = 0.043, η_p_^2^ = 0.154, F_1,25_ = 4.54), and a large effect for a time‐of‐day × sex × limb interaction (*p* = 0.053, η_p_^2^ = 0.142, F_1,25_ = 4.13). Separate follow‐up repeated measures ANOVA tests on each limb showed that there was no time‐of‐day (*p* = 0.402, η_p_^2^ = 0.028, F_1,25_ = 0.726) or between sex (*p* = 0.322, η_p_^2^ = 0.039, F_1,25_ = 1.02) effects for elbow flexor force steadiness. However, the analysis for the knee extensors revealed a time‐of‐day × sex interaction (*p* = 0.013, η_p_^2^ = 0.224, F_1,25_ = 7.21). Simple effects tests revealed that males had significantly lower CoV values in the evening versus the morning (*p* = 0.025, *d* = 0.734) and the evening CoV for males were lower than the evening CoV for females (*p* = 0.032, *g* = 1.19). Figure 4 shows the CoV data across time‐of‐day, sex, and limb.

## 4 DISCUSSION

The purpose of the present study was to examine neuromuscular function in the morning versus the evening, with specific aims to identify differences between limbs and sexes. Although evening superiority for isometric force is well‐documented, there is an absence of data regarding limb‐ and sex‐dependent responses. The main findings shown here demonstrate that the time‐of‐day influence on neuromuscular function is limb‐dependent. The novel findings show: (1) maximal isometric force is greater in the evening for the knee extensors, but not the elbow flexors, (2) there was a small effect for greater maximal muscle excitation in the evening for the biceps brachii, but not the vastus lateralis, and (3) force steadiness was influenced by time‐of‐day and sex.

### 4.1 Maximal force and EMG

The results show increased maximal isometric force in the knee extensors during the evening versus the morning which is in line with previous data (Knaier et al., 2019; Nicolas et al., 2008), while the elbow flexors show no significant time‐of‐day response which does not align with previous research (Gauthier et al., 1997; Zbidi et al., 2016). Nicolas et al. (2008) examined the neuromuscular parameters of the vastus lateralis between morning (06:00 h) and evening (18:00 h) testing sessions and found a 6.8% increase in maximal force from morning to evening which aligns with the current findings. Of the previous studies that utilize maximal isometric contractions, reports show diurnal changes in force ranging from 10% to 12% for the knee extensors (Giacomoni et al., 2005; Guette et al., 2005a) and 14% to 22% for the elbow flexors (Gauthier et al., 1997). Guette et al. (2005a) show that isometric force of the knee extensors for both dominant and the non‐dominant leg increased by 11% in the evening compared to the morning, which aligns with the present data that exclusively utilized the dominant leg and found a mean increase of 10% from morning to evening. Giacomoni et al. (2005) found minimal evidence of time‐of‐day effects for knee extensor force across six different times of the day. Gauthier et al. (1997) tested maximal isometric elbow flexion at six different times of day and found an approximately 14% increase in force in the evening (15:00–18:00 h) compared to morning (06:00–09:00 h) sessions while the current study found only a mean 2% increase in maximal isometric force from morning to evening. In a multi‐day protocol, Sedliak et al. (2008) found an increase in maximal force of the knee extensors from morning to evening over a two‐day period, highlighting the day‐to‐day repeatability of diurnal patterns in muscle force. It is unclear why the influence of time‐of‐day on maximal force was specific to the knee extensors, it is possible that physiological differences in neural innervation and contractile properties explain the contrasting responses, but methodological considerations relating to ambulation through the day may have a role.

The EMG data shows that maximal muscle excitation was opposite of participant maximal force, with the vastus lateralis showing no significant difference in excitation between morning and evening while the excitation of the biceps brachii was slightly higher in the evening than in the morning on average. The majority of participants (~60%) showed greater EMG amplitude values in the evening versus the morning for the biceps brachii but there was considerable interindividual variability and a few high responders to evening testing (Figure 3) so this finding should be interpreted with caution. Nevertheless, the EMG data are interesting as previous work shows a parallel increase in EMG with force in the evening versus the morning (Castaingts et al., 2004; Küüsmaa et al., 2015). However, this is not a consistent finding as other studies have shown no significant time‐of‐day influence on muscle excitation for the biceps brachii (Gauthier et al., 1996) and vastus lateralis (Guette et al., 2005a; Nicolas et al., 2008; Sedliak et al., 2008). Importantly, these studies show that maximal force is generally greater in the evening despite similar muscle excitation levels in the morning and evening. Although neural drive is a determinant of muscle force, potential neural mechanisms to explain diurnal variation in force levels remain elusive. Since others (Gauthier et al., 1996; Gueldich et al., 2016; Martin et al., 1999; Sedliak et al., 2008) have shown that the rate of force development is greater in the evening, we examined several intervals of the force‐ and EMG‐time curves during ballistic intent contraction as an indirect way to infer volitional neural drive since these measurements are largely determined by motor neuron behavior (Del Vecchio et al., 2019). We show no time‐of‐day effect for RFD or RER, though the effect sizes followed a similar pattern as the maximal force values for the respective limbs. There is compelling evidence from transcranial stimulation data that diurnal variations influence GABA‐mediated intracortical inhibition and cortical excitability (Lang et al., 2011; Tamm et al., 2009). Tamm et al. (2009) show that differences in corticospinal excitability levels between the morning and evening depend on the diurnal chronotype of the individual, similar interindividual variation in the time‐of‐day response between diurnal chronotypes has been shown in training studies (Chtourou et al., 2012; Küüsmaa et al., 2016). Nevertheless, our EMG inferences provide little insight into the neural contributions to the present force data.

The diurnal changes in the contractile properties of the muscle may be attributed to intracellular variation of calcium kinetics and excitation‐contraction coupling mechanisms (Edgar & Dement, 1991; Partch et al., 2014), the circadian rhythm in core temperature (Racinais et al., 2004, 2005; Racinais & Oksa, 2010; Taylor et al., 2011), and diurnal variation of circadian clock genes (Douglas et al., 2021). For example, evoked responses from the quadriceps muscles have shown greater twitch torque and rates of twitch torque development in the evening versus the morning (Guette et al., 2005b; Martin et al., 1999). The role of core body temperature is important too as diurnal differences are diminished in warm environments (Racinais et al., 2004) and following extended warmups (Taylor et al., 2011). The increase in maximal muscle strength that occurs with slight elevations (1°C) in central body temperature (Racinais et al., 2004, 2005; Racinais & Oksa, 2010) has been attributed to enhanced contractile kinetics and greater muscle fiber conduction velocity (Shephard, 1984). More recently the discovery of skeletal muscle clock genes has revealed the existence of rhythmic pathways in human skeletal muscle that affect approximately 8% of muscle genes (Dyar et al., 2014). The data show the transcription of human skeletal muscle clock genes enriched genes associated with inflammation, immune responses, myofilament phosphorylation, and mitochondrial activity (Dyar et al., 2014), thus influencing time‐of‐day differences in maximal isometric muscle force (Partch et al., 2014; Takashima, 2009). More research is needed on the role that skeletal muscle clock genes may have on altering diurnal muscle performance, but it remains a potential mechanism for time‐of‐day alterations seen in the present study (Douglas et al., 2021; Vaara et al., 2018).

### 4.2 Force steadiness

A novel finding of the present study relates to the sex difference in force steadiness for the knee extensors. This topic has received attention lately and the current data offers new insights. Our findings show that at 30% of maximal isometric force, there were small, nonsignificant effects for lower force steadiness for females compared to males for the elbow flexors in the morning (*g* = 0.303) and evening (*g* = 0.396). However, the force steadiness during knee extension was influenced by sex and time of day. More specifically, males showed improvements in force steadiness from morning to evening and the magnitude of force variability in the evening was significantly lower than the females. To our knowledge, there is no other data examining force steadiness in the morning versus the evening, so interpretations are challenging. However, in a thorough review, Jakobi et al. (2018) outline several factors that should be considered when interpreting sex‐based differences in force steadiness. The size and type of motor units, the firing behavior of the active motor units, agonist versus antagonist control, absolute muscle strength, and tendon properties. Of these, recent motor unit recordings during steady contractions show evidence of greater coefficients of variation of the inter‐pulse interval of the motor unit firings and greater incidence of doublet discharges across a range of intensities in the tibialis anterior muscle in females compared to males (Inglis & Gabriel, 2021). Although the present data is without motor unit recordings, it is nonetheless intriguing that sex‐based differences in force steadiness were more pronounced in the evening than the morning and for the lower than the upper limb. These findings may lend support to the influence of diurnal catecholamine releases on motor neuron firing properties (Pereira et al., 2015) and the size of the active motor units on force steadiness (Brown et al., 2010; Harwood et al., 2010; Jakobi et al., 2018).

### 4.3 Sex comparisons

A major aim of this study was to determine if time‐of‐day changes in neuromuscular performance are influenced by sex. A recent meta‐analysis shows that of the research examining time‐of‐day effects on short‐term performance, approximately 10% of subjects are female (Mirizio et al., 2020). Since females exhibit a relatively greater flux of the gonadal steroid hormones (Bailey & Silver, 2014), it was reasoned that this may influence the diurnal pattern of neuromuscular function. The present findings of sex differences in force steadiness in the evening for the knee extensors indicate that diurnal variation between males and females may present during submaximal, not maximal tasks. In contrast to our hypothesis, the data generally indicate greater variability between morning and evening for males compared to females. Sex differences in gonadal steroid hormone receptor density in the suprachiasmatic nucleus as well as its major afferent pathways are shown in animal models (Bailey & Silver, 2014; Kuljis et al., 2013). These observations represent a direct mechanism for sex‐based differences in diurnal variation, yet the paucity of data in both animals and humans render limited speculations on the functional outcomes that may result (Bailey & Silver, 2014; Kuljis et al., 2013).

### 4.4 Limitations

Certain limitations should be noted. In the present study, participants were not matched into groups based on their respective diurnal chronotypes, there was no assessment of participant arousal levels, and neurohormonal data was not collected. For the female group, the menstrual cycle phase in which testing occurred was standardized within but not between participants. Although menstrual phase has shown little influence on maximal force (Ansdell et al., 2019) the potential heterogeneity from phase‐specific time‐of‐day effects (Bambaeichi et al., 2004; Birch & Reilly, 2002), hormonal contraceptive use, and contraceptive types within our female sample may have introduced variance that should be considered. Despite within‐participant maximal EMG comparisons, it is important to emphasize that our EMG measurements were not normalized to the compound muscle action potential, but to the maximal isometric contraction which limits mechanistic interpretations of peripheral‐central contributions. However, the EMG rise data were normalized to the maximal EMG amplitude values. Lastly, the inability to measure evoked twitch properties also limits the peripheral interpretations that may explain the present force data.

## 5 CONCLUSIONS

The present study shows diurnal variation in maximal isometric force is limb‐dependent, with greater isometric forces in the evening for the knee extensors but not the elbow flexors. The EMG responses did not parallel the force data, resulting in non‐uniform diurnal force and muscle excitation between upper and lower limbs. This study also outlines diurnal sex differences in force steadiness. In general, females show less susceptibility to diurnal variation in neuromuscular function than males in the present study. Future research is needed to identify whether these limb‐dependent diurnal responses have implications for optimizing neuroplasticity during exercise training and rehabilitation and other investigations should continue to examine how sex influences diurnal variation in exercise performance as there is a clear biological gradient (Bailey & Silver, 2014; Kuljis et al., 2013; Lang et al., 2011; Tamm et al., 2009) but a substantial lack of data.

## CONFLICT OF INTEREST

We report no conflicts of interest.

## ETHICAL APPROVAL

Ethics approval for this study was granted by the Institutional Review Board for Human Subjects Research at Texas Christian University. Participants were provided verbal and written explanations of the risks associated with the experimental protocol before obtaining written informed consent.

## AUTHOR CONTRIBUTIONS

Garrett R. Augsburger and Joshua C. Carr conceived and designed research; Garrett R. Augsburger and Alisa Soloveva performed experiments; Garrett R. Augsburger, Alisa Soloveva, and Joshua C. Carr analyzed data; Garrett R. Augsburger, Alisa Soloveva, and Joshua C. Carr interpreted results; Garrett R. Augsburger drafted the manuscript; Garrett R. Augsburger, Alisa Soloveva, and Joshua C. Carr edited and revised the manuscript; Garrett R. Augsburger, Alisa Soloveva, and Joshua C. Carr approved the final version of the manuscript.
